# Caveolin as a potential drug target for cardiovascular protection

**DOI:** 10.3389/fphys.2012.00280

**Published:** 2012-07-18

**Authors:** Stephanie L. Sellers, Andy E. Trane, Pascal N. Bernatchez

**Affiliations:** Department of Anesthesiology, Pharmacology and Therapeutics and The James Hogg Research Centre, University of British ColumbiaVancouver, BC, Canada

**Keywords:** caveolin-1, caveolin-3, caveolae, cardioprotection, nitric oxide, therapeutics, cardiovascular disease

## Abstract

Caveolae and caveolin are key players in a number of disease processes. Current research indicates that caveolins play a significant role in cardiovascular disease and dysfunction. The far-reaching roles of caveolins in disease and dysfunction make them particularly notable therapeutic targets. In particular, caveolin-1 (Cav-1) and caveolin-3 (Cav-3) have been identified as potential regulators of vascular dysfunction and heart disease and might even confer cardiac protection in certain settings. Such a central role in vascular health therefore makes manipulation of Cav-1/3 function or expression levels clear therapeutic targets in a variety of cardiovascular related disease states. Here, we highlight the role of Cav-1 and Cav-3 in cardiovascular health and explore the potential of Cav-1 and Cav-3 derived experimental therapeutics.

## Introduction

Caveolae, less commonly termed plasmalemmal vesicles, were first discovered by Yamada (Yamada, 1955) and Palade (Palade, 1953). They are defined as a specialized type of lipid raft that typically take on the appearance of 50–100 nm flask-shaped invaginations in the plasma membrane. While present in most tissues, the presence of caveolae is most notable in adipocytes, endothelial cells, fibroblasts, muscle tissue, and epithelial cells. The main physiological roles of caveolae are in regulating and facilitating cell signaling, specific types of vesicular transport such as endocytosis, and lipid content of the plasma membrane. Caveolin, the coat protein of caveolae, are a family of 22–24 kDa proteins with cytosolic N and C termini with three known isoforms: caveolin-1 (Cav-1), caveolin-2 (Cav-2), and caveolin-3 (Cav-3) (Krajewska and Masłowska, 2004; Chidlow and Sessa, 2010; Hansen and Nichols, 2010).

Cav-1 and Cav-2 are predominant in the cardiovascular system. Cav-1 is required for caveolae formation and maintenance in non-muscle-based cell types and has prominent expression in endothelial cells. In the cardiovascular endothelium, Cav-1 is a key regulator of nitric oxide (NO) by endothelial nitric oxide synthase (eNOS), calcium, and vascular growth and remodeling. Notably, although the expression pattern of Cav-1 is similar to that of Cav-2, Cav-2 is not required for caveolae formation. However, Cav-2 is thought to influence caveolae formation as a result of hetero-oligomerization with Cav-1 (Galbiati et al., 1998; Drab et al., 2001; Razani et al., 2002; Sowa et al., 2003; Bauer et al., 2005; Bernatchez et al., 2005; Yu et al., 2006; Lay et al., 2009). Meanwhile, unlike Cav-1 and Cav-2, Cav-3 is expressed predominately in muscle. This includes in vascular smooth muscle as well as cardiac and skeletal muscle tissue where it is essential for caveolae formation (Song et al., 1996; García-Cardeña et al., 1997).

Caveolae, and/or its components including caveolins are linked to disease including atherosclerosis (Frank et al., 2009), cancer (Goetz et al., 2008), bowel disease (Andoh et al., 2001), lipid disorders (Lay et al., 2009; Rahman and Swärd, 2009), cardiac disorders (Xu et al., 2008), and respiratory disease (Gosens et al., 2008). Despite being associated with so many pathologies, caveolins also appear to have a role in cardiac protection. This presents caveolins as both a therapeutic target for fighting disease by understanding their role in disease pathogenesis and protecting health by elucidating their contribution to vascular protection. Herein, we aim to bring focus to the therapeutic potential of caveolins by first highlighting the role of caveolins in cardiovascular disease and cardiac protection, and then reviewing the current state of development of caveolae or caveolin-based cardiovascular therapeutics. To do so and to appreciate the diverse role of caveolin, the therapeutic implications of both Cav-1 and Cav-3 are explored. In both cases, we provide an overview of the noted roles both caveolin isoforms play in cardiovascular disease and protection. Subsequently, we look at the potential therapeutic benefits of targeting caveolin, how such targeting is being achieved, and the potential clinical outcomes.

## Cav-1, nitric oxide and vascular disease

Cav-1 is the predominant caveolin isoform in the cardiovascular system, with a particularly notable role in endothelial cells as Cav-1 drives the formation of caveolae and is also required for maintenance within the plasma membrane (Gratton et al., 2004; Sowa, 2012). The requirement of Cav-1 for caveolae formation was first indicated by studies in which Cav-1 was expressed in lymphocytes, which do not normally exhibit caveolae, and subsequently resulted in caveolae genesis (Fra et al., 1995). The requirement of Cav-1 for caveolae maintenance is evidenced by studies depleting Cav-1 wherein loss of Cav-1 results in loss of caveolae (Galbiati et al., 1998). Furthermore, the importance of Cav-1 in vascular function has been elucidated by the development of murine models. For example, the homozygous deletion of the Cav-1 gene in mice was shown to produce viable offspring. However, a variety of cardiovascular phenotypes were observed including elevated NO levels, impaired angiogenesis, cardiomyopathy, and pulmonary hypertension (Lay and Kurzchalia, 2005). Additionally, Cav-1-null animals were also found to be protected against atherosclerosis (Frank et al., 2004), while overexpression of Cav-1 in the endothelial layer was found to accelerate the progression of atherosclerosis (Fernández-Hernando et al., 2010).

The above examples demonstrate a notable role for Cav-1 in a number of vascular diseases phenotypes, and expose Cav-1 as a notable therapeutic target for protection from vascular disease. However, the first step to developing Cav-1 targeted therapeutics is to understand the means by which caveolin is involved in cellular processes leading to disease states. One of the main ways Cav-1 regulates cellular signaling is through a sequence known as the caveolin scaffold domain (CSD). The CSD consists of residues 82–101 of Cav-1 (^82^DGIWKASFTTFTVTKYWFYR^101^) and controls cellular signaling by binding and sequestering proteins via a motif known as the caveolin binding sequence. This sequence is фxфxxxxфxxф, where “ф” is an aromatic amino acid, and “x” is any amino acid and binds a number of proteins. Caveolae-related proteins are diverse in nature, ranging from G-protein coupled receptors to tyrosine kinases and signaling enzymes, and hence mediate a host of cellular effects as reviewed previously (Okamoto et al., 1998; Roth and Patel, 2011). One of the key enzymes bound by Cav-1 that is a major target of caveolin-based therapeutics is nitric oxide synthase (NOS). NOS is a regulator of vascular health and disease through the production of the vasodilatory gas NO. Constitutive NO within the vasculature is produced by eNOS, which catalyzes the conversion of L-arginine to L-citrulline and NO (Albrecht et al., 2003; Minshall et al., 2003; Sessa, 2004, 2005). However, when bound to the CSD, eNOS is held in an inactive state, thereby limiting NO production. The proposed site of binding of eNOS to Cav-1 is within the catalytic domain of eNOS where there is a conserved caveolin-binding motif (**F**SAAAP**F**SG**W**). This motif is the proposed on/off switch for eNOS due to the finding that calmodulin, an activator of eNOS, has been shown to compete with Cav-1 for this binding site and thereby regulate NOS activity. Indeed, site-directed mutagenesis within eNOS prevents binding of Cav-1 and mutation within the CSD of Cav-1 mitigates its control of eNOS activity *in vivo*. Furthermore, in addition to competing with calmodulin for the caveolin-biding motif, Cav-1 also dynamically regulates the ability of the serine/threonine amino acid kinase Akt to access eNOS. This effectively allows Cav-1 to further govern eNOS activity, as Akt is essential for regulating eNOS phosphorylation at Ser^1179^ and Thr^497^, which dictate the level of eNOS activity (Couet et al., 1997; García-Cardeña et al., 1997; Michel et al., 1997).

Though complex, the regulatory setup of Cav-1/eNOS is important. This is because NO is central to normal physiological processes and altered regulation of NO is commonplace in the setting of vascular diseases. Changes in NO regulation contributing to pathophysiological processes, termed endothelial dysfunction, is clinically characterized by reduced NO bioavailability and is associated with worse cardiovascular outcomes (Widlansky et al., 2003). For example, impaired flow-dependent endothelium mediated dilation of the radial artery has been reported to be an independent predictor of cardiac-related mortalities in human studies (Fischer et al., 2005). Additionally, the importance of NO in endothelial function has also been suggested in animal studies. Felines that underwent left anterior descending coronary artery occlusion and subsequent reperfusion had reduced endothelial function that progressively declined with greater reperfusion times. Furthermore, necrotic areas and at risk areas increased with reperfusion times (Tsao et al., 1990). Interestingly, the use of NO donors SIN-1 or C87-3754, in the same feline model, led to reduction of both necrotic area and endothelial dysfunction following reperfusion (Siegfried et al., 1992). Similarly, in a canine model of bypass surgery, the use of vardenafil, a phophodiesterase-5 inhibitor, which prevents the breakdown of NO-mediated cGMP accumulation, showed increased cardiac and endothelial function (Szabó et al., 2009). Beyond endothelial dysfunction, the pathophysiological role NO is also seen in the setting of pulmonary hypertension. Both humans and Cav-1 deficient mice are noted to develop pulmonary hypertension as a result of uncontrolled eNOS activation due to a lack of Cav-1 (Zhao et al., 2009). Similar constitutive hyper-activation of eNOS is also known to drive cardiomyopathy seen in Cav-1 deficiency (Wunderlick et al., 2008). Overall, the studies considered above demonstrate that *in vivo* Cav-1 plays a vital role in NO-based pathophysiology and thereby support the use of NO-based therapies to improve cardioprotection by targeting Cav-1. Therefore, to further examine therapeutic potential and applications, therapies regulating NO in vascular disease and cardioprotection are considered below as well as how this may be best achieved through Cav-1-based therapeutics and other clinical benefits of targeting Cav-1.

## Nitric oxide and caveolin-1 therapeutics

A variety of pharmacological substances have been utilized clinically to promote cardiac protection during ischemia. These include betablockers, glucose-insulin-potassium infusion, sodium-hydrogen exchange inhibitors, adenosine, calcium channel blockers, and K_ATP_ channel openers. However, the level of success has been mixed and the search for improved therapies has continued (Kloner and Rezkalla, 2004). Therapies involving NO targeting may be one such “bigger and better” approach. Generally, therapeutics employed to increase NO typically consists of delivering pre-cursors (e.g., L-arginine), mimetics, donors (e.g., glycerol trinitrate and NONOates), or hybrid compounds (e.g., NO-linked compounds) systemically (Megson and Webb, 2001). Unfortunately, the clinical outcomes of such therapeutics are varied. A meta-analysis of the use of nitroglycerin and nitroprusside (NO donors) in acute myocardial infarct suggested that nitrates reduced mortality be around a third (Yusuf et al., 1988). Conversely, more recent studies failed to identify any benefits or improved clinical outcome associated with long-term nitrite usage (Group, 1994; Yamauchi et al., 2008), whilst another study suggested that nitrate therapy subsequent to acute coronary events increased mortality (Nakamura et al., 1999). However, while the effect on mortality have varied significantly, NO targeted therapeutics appear to be cardioprotective. It was found that when patients with acute myocardial infarction were treated with nitroglycerin within four hours of developing chest pain, infarct size was reduced and the left ventricular function was better in comparison to control (Jugdutt and Warnica, 1988). Also, the use of isosorbide mononitrite had a greater protective effect on patients that presented with no Q wave or ST-segment elevation (Morris et al., 1995). Another study found that early usage of nitroglycerin, in conjunction with verapamil, led to smaller infarct sizes in a third of the patients that presented with ST-segment elevation (Beltrame et al., 2002). Furthermore, in the context of coronary artery bypass surgeries, the addition of L-arginine, an NO pre-cursor, into the cardioplegia solution (Carrier et al., 2002) or inhalation of NO was found to reduce biomarkers associated with cardiac damage, such as troponin, indicating that supplementation with NO-based therapies was cardioprotective (Gianetti et al., 2004). Therefore, study results support further development and exploration of NO-based therapeutics overall.

One aspect of NO-based therapeutics that may contribute to variability in results of NO therapeutics is the limitations and side effects associated with exogenous sources of NO. Clinical use of nitrates have demonstrated a variety of side effects including dizziness, headaches, and hypotension, all of which are linked to the vasodilatory effects of nitrates (Thadani and Ripley, 2007). Furthermore, long-term use of organic nitrates, such as nitroglycerin, can promote tolerance. This means that subsequent and continued treatment requires greater doses, which subsequently promotes eNOS uncoupling. Detrimentally, this in turn leads to greater levels of superoxide production and has clear implications on the long term clinical usage of such compounds (Gori and Parker, 2002; Parker, 2004). Therefore, the development of compounds capable of chronically elevating endogenous NO levels via eNOS or relevant enzymes may prove to be of a greater therapeutic benefit, as current therapeutics deliver high boluses of NO that may be harder to regulate and produce more side effects.

**Figure 1:**
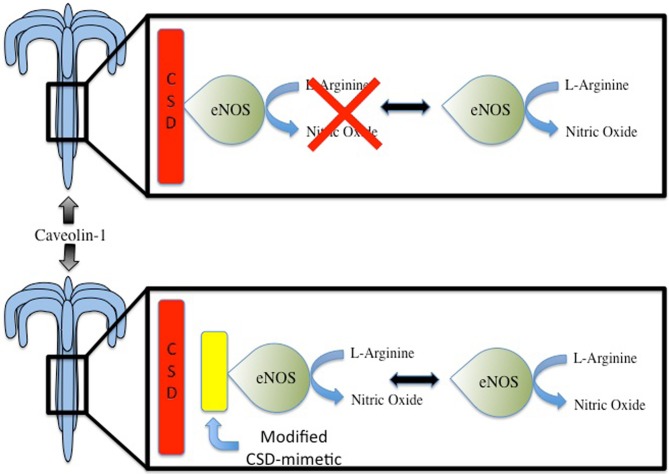
**Under basal conditions, endothelial nitric oxide synthase (eNOS) is the main producer of nitric oxide (NO) in the cardiovascular system.** Interaction between eNOS and Caveolin Scaffolding Domain (CSD) of Caveolin-1 leads to inhibition of eNOS activity. Compounds that can mimic the Cav-1 CSD may potentially disrupt the inhibitory interaction, thereby leading to increased NO production. This may have potential benefits in the context of cardiac protection, which seemingly is, in part, mediated by NO.

A promising approach to increase basal levels of NO via Cav-1 targeting would be to directly disrupt the inhibitor interaction between eNOS and Cav-1. This interaction has been validated in *in vitro* and *in vivo* models as direct interaction between eNOS and Cav-1 and documented through a variety of techniques including yeast two-hybrid, glutathione-S-transferase pulldown and co-immunoprecipitation (Ju et al., 1997; Gratton et al., 2000). As a result of characterizing the interaction of eNOS and Cav-1, a cell permeable peptide containing the Cav-1 CSD was developed and was successful in decreasing eNOS activity and subsequently reducing vascular leak (Bucci et al., 2000; Fulton et al., 2001; Minshall et al., 2003). Furthermore, peptides based on residues 82–101 of the Cav-1 CSD were found to be able to inhibit eNOS activity (Ju et al., 1997). Moreover, use of cell-peptide fragments based off of the Cav-1 CSD have allowed for further probing of the inhibitory mechanism of the CSD on eNOS activity and refinement of Cav-1 therapeutic targets. Specifically, it was found that residues ^90^Thr, ^91^Thr, and ^92^Phe in particular played essential roles in Cav-1/eNOS interaction. In fact, use of a full-length Cav-1 mutant with a single alanine substitution in lieu of ^92^Phe entirely abolished Cav-1's inhibitory effects on eNOS (Bernatchez et al., 2005). Furthermore, not only does the substitution of ^92^Phe with alanine in Cav-1 prevent eNOS inhibition, it also promotes NO and reduces superoxide release. Notably, this was accomplished without disrupting the basic biochemical properties of Cav-1 and eNOS. These findings then lead to the development of a cell-permeable Cav-1 CSD with alanine substitution of residues 90–92 that was able to induce vasorelaxation of aortic rings and the reduce blood pressure of mice in an eNOS-dependent manner (Bernatchez et al., 2011). Notably, this was the first study to highlight the potential of modified Cav-1 CSD-derived peptides as antagonists to promote NO release and is an important step forward in advancing Cav-1 targeted therapeutic for regulating NO for cardioprotective purposes (Figure 1). However, as with all therapeutics, there may be inherent limitations which are the focus of future studies. A final approach to consider for regulating NO through interrupting Cav-1 binding of eNOS is through other pharmacological compounds. One such compound is amlodipine, a calcium channel blocker. Amlodipine is noted to increase production of NO in endothelial cells and this was subsequently found to be capable of increasing NO release by inhibiting Cav-1 binding of eNOS in cultured endothelial cells (Batova et al., 2006; Sharma et al., 2011).

## Other targets of Cav-1 and cardiovascular therapeutics

Although targeting of the Cav-1 CSD has the most potential for impact on eNOS regulation, as evidenced by studies which showed no effects of a Cav-1 CSD peptide in eNOS knockout mice, the scaffold nature of Cav-1 also presents the potential for Cav-1 CSD peptides to regulate vascular disease and cardioprotection through other proteins which bind the CSD (Gratton et al., 2003; Roth and Patel, 2011). For example, injection of Cav-1 peptides into isolated perfused rat hearts led to the preservation of left ventricular function post-ischemic reperfusion (Young et al., 2001). The authors noted reduced immune cell adherence and increased NO production, which they had attributed to the inhibition of PKC, a Cav-1 regulated protein that inhibits eNOS activity and promotes superoxide release (Hirata et al., 1995; Zulueta et al., 1995). Furthermore, other mediators of vascular disease that interact with the Cav-1 CSD present alternative therapeutic avenues. Notably, Cav-1 deficient mice have demonstrated cardiac hypertrophy owing to hyperactivation of ERK 1/2 signaling under basal conditions (Cohen et al., 2003). Similarly, another study found that Cav-1 deficient mice displayed dilated left ventricles and right ventricular hypertrophy (Zhao et al., 2002), and in mice that had undergone left anterior descending coronary artery ligation, those lacking Cav-1 failed to increase β-adrenergic receptor density, leading to reduced cAMP production, PKA phosphorylation and survival (Jasmin et al., 2011). Additionally, many cardiac ion channels (e.g., Kv1.5, Nav 1.5, and HCN4) responsible for normal cardiac function are targeted to caveolae (Maguy et al., 2006). However, with regards to cardioprotection, mitochondrial ion channels play a more significant role in the determination of cell fate (O'Rourke et al., 2005; Nishida et al., 2010). Disruption of caveolae with methyl-beta-cyclodextrin led to decreased eNOS-dependent signaling and nitrosylation of mitochondrial proteins, which was associated with the lost of ischemic preconditioning (Sun et al., 2012).

Notably, however, Cav-1 does not always act independently of other caveolin isoforms. Cav-1 has also been suggested to be able to form heteromeric complexes with Cav-3 in atrial cardiac myocytes, which was responsible for apoptosis in response to doxorubicin, a chemotherapeutic agent (Volonte et al., 2008). Overall, Cav-1 in cardiac myocytes is not as well characterized as Cav-3; however, taken together, the facts suggest that Cav-1 plays a functionally important role in cardic function and protection as a mediator of cellular signaling. Hence, CSD-derived peptides from Cav-1 may elicit interesting responses in the context of cardioprotection, but more studies are required to confirm such speculation. Furthermore, while all of findings highlighted regarding the potential for targeting Cav-1 are promising, it is important to consider the potential pitfalls of targeting Cav-1 given the complex and diverse role of Cav-1 *in vivo*. These potential consequences include off-target effects such as non-specifically inhibiting many of the possible Cav-1 signaling events instead of a single key event as intended. This is turn may have detrimental outcomes on normal cell physiology and speaks to the need to consider the widespread effects of Cav-1 targeted therapeutics and importance of developing highly specific targets within Cav-1.

## Caveolin-3 in cardiovascular disease and protection

Cav-3 is essential for formation of caveolae in cardiac and skeletal muscle and is the dominant caveolin isoform in cardiomyocytes (Song et al., 1996). Given the critical role of caveolins in cell physiology and the localization of Cav-3 in cardiac muscle, it is of little surprise that Cav-3 is a player in both cardiac disease and protection. The major pathological cardiovascular features associated with change in Cav-3 function are those leading to hypertrophy and myopathy, which are hallmarks of cardiovascular disease. Notably, while Cav-3 is also a key player in types of muscular dystrophy, which can have cardiovascular components (Catteruccia et al., 2009), this pathological role of Cav-3 is outside of the scope of this review and will not be considered further. However, the vital role of Cav-3 in ischemia preconditioning will be explored. Ischemic preconditioning, which imbibes cardiomyocytes with the ability to resist damage and injury during subsequent ischemic injury events has strong therapeutic potential. Such Cav-3 dependent preconditioning involves complicated and often controversial signaling cascades. In fact, signaling cascades mediating Cav-3 involvement in cardiovascular pathogenesis and cardioprotection, have been reviewed elsewhere recently (Roth and Patel, 2011). Therefore, in order to bring new perspective, focus, and insight to Cav-3 in vascular health, we subsequently examine the relevance and development of Cav-3-based therapeutics. In doing so, the current understanding of and potential of Cav-3 therapeutics in offering cardioprotection in the classic sense, from ischemia, and protection from development of cardiovascular disease is presented.

## Protection from ischemia

Beyond contributing to the pathophysiology of a number of diseases, caveolins are notable in facilitating cardiac protection in myocardial ischemia-reperfusion injury. Protection from ischemia-reperfusion injury is often provided experimentally via ischemia preconditioning and was first noted in the 1980's (Murray et al., 1986). This involves induces brief periods of ischemia repeatedly, which induces an intrinsic protection from subsequent ischemia-reperfusion. Interestingly, cardioprotection from ischemic events can also be achieved through exposure to a number of already known compounds. These include opioids such as morphine (Schultz et al., 1996), and anesthetics including isoflurane (Cason et al., 1997; Kersten et al., 1997). In all cases of induced ischemia precondition (ischemia, opiods, and anthesthics) the process has been found to be dependent upon Cav-3. This is highlighted in mice lacking Cav-3, which have an inability to be preconditioned to ischemic injury through ischemia preconditioning, or preconditioning treatment with isofluorane, or opiods (Horikawa et al., 2008; Tsutsumi et al., 2010). Therefore, given the central role of Cav-3 in ischemic preconditioning, Cav-3 presents itself as an interesting therapeutic target for inducing cardioprotection from ischemia.

Unfortunately, targeting of the Cav-3 dependent preconditioning using the methods of ischemia, opiods, or anesthetics exposure does not appear to be a viable therapeutic option for translation from bench to beside. This is due to the perceived difficulties in implementing such treatments and the risk of severe adverse complications. Fortunately, a number of alternative approaches taking advantage of the direct link between Cav-3 and cardioprotection from ischemia are being developed and identified. For example, while previously in the article we considered the use of peptides in targeting Cav-1 regulation of eNOS, thereby altering basal NO levels to aid cardioprotection, peptides are also being used to target Cav-3. Recently, Shen and colleges (2011), utilized a Cav-3 CSD peptide to induce protection against apoptosis in cardiomyocytes. Following the finding that hypoxic cardiomyocytes from rats that were subsequently reoxygenated had oxidative damage and decreased Cav-3 levels, the authors demonstrated that use of a Cav-3 peptide of the scaffold domain was able to eliminate oxidative damage. In this case, protection from oxidative damage was attributed to inhibiting production of O_2_^−^, increased superoxide dismutase activity (SOD), and inhibition of the caspase-3. Interestingly, this is also an example of successful use of a Cav-1 peptide for cardioprotection as Shen and colleges also found Cav-1 peptides to be protective. However, Cav-1 peptides were less effective than Cav-3 peptides in preventing oxidative damage.

Although the use of Cav-3 peptide does show potential, other therapeutic interventions to allow for overexpression of Cav-3 are being perused. Adenovirus is one such intervention. Indeed, the cardioprotective effect of overexpression of Cav-3 via adenovirus has been noted by Tsutsumi and colleges (2008). Transfection of cardiac myocytes increased caveolae number, and Akt and GSK3β levels, which was previously been noted to be important in mediating preconditioning to ischemia (Hausenloy et al., 2005). Additionally, the protective effects against ischemia/reperfusion injury seen in Cav-3 adenovirus treated myocytes were also seen in transgenic mice that had myocyte-specific overexpression of Cav-3. These mice had decreased infarct size following injury attributable to enhanced Akt and GSK3β phosphorylation. Subsequently, this allowed the mice to respond to injury similar to wild-type mice which had been preconditioned. Clearly, these findings are a promising beginning for use of adenovirus to alter Cav-3 levels, and although there will undoubtedly be a number of obstacles in moving them forward to therapeutic applications, the use of adenoviruses is also being pursued in a number of other fields including treatment of cancer, diabetes, and peripheral artery disease as highlighted in recent publications (Creager et al., 2011; Tang et al., 2011; You et al., 2011), which will serve to expedite the process of moving adenovirus-based treatment from bench to bedside.

Despite the exciting advances in beginning to develop therapeutics targeted at Cav-3 as discussed above, perhaps the answer is in pharmaceuticals and/or other compounds that already exist, but have not been considered in the light of a potential Cav-3 modulator. One an example of these is tocotrienols. Members of the vitamin E family, tocotrienols, have antioxidant properties and have been studied for use in cancer and have noted benefits in cardioprotection (Qureshi et al., 1991; Srivastava and Gupta, 2006). However, it is only recently that evidence demonstrating that tocotrienols mediate cardioprotection by differentially regulating Cav-1 and Cav-3 binding partners such as MAP kinases, HO-1 and eNOS, leading to elevated pro-survival signaling (Das et al., 2008). Ceramide has also been noted in preconditioning to ischemic injury in relation to caveolin. During preconditioning there is a notable increase in ceramide and sphingosine (Cui et al., 2004). Furthermore, increased p38MAPKα/Cav-1 interaction and decreased p38MAPKβ/Cav-3 interaction was observed following ischemia reperfusion of preconditioned hearts, helping to promote pro-survival signals and preserve heart function (Das et al., 2007). Clearly, the two examples above are far from therapeutic use and would most likely (1) have notable systemic and off target effects and (2) require more work to make their effects more specific. While these points on need for further development and refinement are true for all caveolin targeted therapeutics, these examples are representative of the concept that compounds and drugs that have already been developed may hold untapped potential in modulating caveolin-mediated activities and therefore, be useful in cardioprotection.

## Protection from cardiovascular disease

Normal heart physiology and vascular function is frequently disrupted and thereby gives rise to a multitude of pathological states. What is interesting about this from a caveolin research point of view is that Cav-3 is a significant player in a number of these diseased states. For example, changes in Cav-3 expression levels are noted in chronic hypoxia, hypertension, and models of heart failure including those characterized by cardiomyocytes hypertrophy and ventricular dysfunction (Shi et al., 2000; Woodman et al., 2002; Ohsawa et al., 2004; Lee et al., 2006; Feiner et al., 2011). Furthermore, given the grave burden of cardiovascular disease on the global population today and the role of Cav-3 in heart physiology, considering how Cav-3 targeted therapeutics may enable cardioprotection in the non-classical sense of preventing or reducing pathogenic changes in the heart and vasculature is important.

In pursuit of the goal of enabling protection from pathological events by targeting Cav-3 a number of therapeutic approaches have been used. Interestingly, many of the approaches overlap with those used to regulate Cav-3 for induction of cardioprotection as reviewed above. One such example is the use of adenovirus. Cardiomyocytes transfected with adenovirus encoding Cav-3 were protected from hypertrophy induced through phenylephrine and endothelin-1 exposure as a result of preventing ERK 1/2 phosporylation. Furthermore, adenovirus encoding a mutant form of caveolin seen in limb-girdle muscular dystrophy had the opposite effect: increased hypertrophy of cardiomyocytes. Therefore, this study not only shows that using adenovirus to alter Cav-3 expression in cardiac myocytes may be a promising avenue of therapeutics, it is also a useful tool in studying deleterious mutations in Cav-3 (Koga et al., 2003). Moreover, regulation of Cav-3 via adenovirus in myocytes was also recently successfully used to increase Cav-3 levels and regulate atrial natriuretic peptides, which are known to be key mediators of cardiac hypertrophy (Horikawa et al., 2011). In addition, a recent study found that inhibition of Cav-3 localized L-type Ca^2+^ channels reduced Ca^2+^ transients and hypertrophic NFAT translocation without reducing contractility, highlighting the potential of targeting caveolae specific signaling responses (Makarewich et al., 2012). Notably, similarity in therapeutic approaches is also seen between Cav-1 and Cav-3. This is because, like the role of Cav-1 in pathogenesis, Cav-3 is also noted to mediate changes in NO (Roth and Patel, 2011). Therefore, altering NO levels to combat cardiac hypertrophy and heart failure has also been explored. Although not directly targeted at Cav-3, LA419, a novel NO donor, was able to prevent cardiac hypertrophy caused by pressure overload in rats by successfully increasing endogenous NO levels. This was the result of increasing interaction between eNOS and heat shock protein 90 (Hsp90) and return of Cav-3 to levels seen during homeostasis (Ruiz-Hurtado et al., 2007). Furthermore, the significant role of Cav-3 in regulating NO within vascular disease extends to muscular dystrophy. Mice with overexpression of a P104L mutant of Cav-3, a model of autosomal dominant limb-girdle musclar dystrophy, showed mislocalization of Cav-3 and development of hypertrophic cardiomyopathy. Upon investigation, it was determined that this was associated with increased eNOS activity and showed that deregulation of Cav-3 within the heart can result in hypertrophic cardiomyopathy (Ohsawa et al., 2004). Alternatively, exploring compounds, that were previous explored for use in therapeutic in contexts other than Cav-3 regulation, is a new point of interest. A recent example of this is estrogen as 17beta-estradiol was found to prevent cardiomyocyte hypertrophy in ovariectomized rats exposed to abdominal aortic contraction by upregulating Cav-3 expression and reducing phospho-ERK1/2. Furthermore, in the same study, the authors found that angiotensin II (Ang II) mediated cardiac hypertrophy could be mitigated by 17beta-estradiol, but the effect was abolished following administration of methyl-beta-cyclodextrin, which disrupts caveolae (Cui et al., 2011). However, while these are promising means of targeting Cav-3 and caveolae, exploring their risks and possible complications will be important for future studies.

## Conclusion

Caveolins are important regulators of a multitude of cardiovascular related events. Specifically, Cav-1 is a key player in cardiovascular health. Of its roles, the regulation of eNOS and thereby of basal NO levels is a vital component of vascular health. The importance of this is illustrated by that fact that a reduction in systemic NO levels has been associated with cardiovascular mortalities and NO levels play important roles in vascular dysfunction. However, it should be noted that findings on mortality following administration of NO have varied. In contrast, studies using NO to promote cardioprotection in events such as bypass surgery and cardiovascular events generally indicate a therapeutic advantage to NO. However, there are limitations with the current methodologies of increasing systemic NO in a bolus manner. Therefore, increasing basal NO appears to be a more attractive solution as it may circumvent current problems associated with nitrates such as tolerance and hypotension. One method of doing so is to develop compounds to directly target the interaction between eNOS and Cav-1 and thereby prevent or limit inhibition eNOS by Cav-1. In this pursuit, peptides of Cav-1 CSD show promise in being able to regulate NO production. Furthermore, targeting of Cav-1 to regulate cellular signaling outside of eNOS is also a front of intense research. Similar to Cav-1, Cav-3 plays many important roles in cardiomyocyte signaling and cardiac function. Peptides generated from Cav-3 are thought to contribute to protective preconditioning of the heart while mutations in endogenous Cav-3 have been associated with cardiac myopathies. Furthermore, several pro-cardiac survival compounds have been demonstrated to have their effects associated with Cav-3 regulations (Table 1). Overall, compounds capable of regulating either Cav-1 and/or Cav-3 possess potentially important therapeutic implications in the context of cardiac health and disease. However, while caveolin targeted therapeutics have great potential, due to complex signaling pathways associated with caveolins they require further development to ensure specificity in order to reach clinical applicability.

**Table 1:** **Recent caveolin-based therapeutics**.

**Therapeutic**	**Target**	**Outcomes**
Peptide	Caveolin-1 scaffold domain	Prevents eNOS inhibition Promotes NO Release Reduces Superoxide Preserves left ventricular function post-ischemic reperfusion
	Caveolin-3 scaffold domain	Protects cardiomyocytes from oxidative damage Increase SOD activity Inhibition of caspase-3 Decreased O_2_^−^
Adenovirus	Overexpression of Cav-3 in cardiomyocytes	Protection from ischemic injury Increased caveolae number Increased Akt and GSK3β levels Decreased infarct size Protection from induced cardiomyocytes hypertrophy Prevention of ERK phosphorylation Increased atrial natriuretic peptides
Pharmaceutical compound	Tocotrienols	Cardioprotection via regulation of cav-1/3 interaction with MAPK, HO-1, and eNOS
	17β-estradiol	Prevents induced cardiomyocytes hypertrophy Increased cav-3 Decreased ERK Prevents Ang II mediated hypertrophy
	LA419	Prevents pressure-overload induced hypertrophy Increased endogenous NO

### Conflict of interest statement

The authors declare that the research was conducted in the absence of any commercial or financial relationships that could be construed as a potential conflict of interest.
